# Trp53 inactivation in the tumor microenvironment promotes tumor progression by enhancing pro-inflammatory tumor stromal populations

**DOI:** 10.1186/2051-1426-1-S1-P189

**Published:** 2013-11-07

**Authors:** Gang Guo, Luis Marrero, Augusto Ochoa, Yan Cui

**Affiliations:** 1 Sanely Scott Cancer Center, LSUHSC-NO, New Orleans, LA, USA; 2 Microbiology, Immunology, and Parasitology, LSUHSC-NO, New Orleans, LA, USA

## 

Inactivation of the tumor suppressor p53 is one of the leading causes of cancer as *p53* inactivation via somatic mutations occurs in 50% of human cancers and sometimes in fibroblasts within the tumor microenvironment (TME). Recent studies by our laboratory and others suggest that *p53* inactivation promotes a pro-inflammatory host microenvironment - elevated serum inflammatory cytokines/chemokines, enhanced Th17 cells, and augmented differentiation of myeloid cells, including myeloid derived suppressors (MDSCs). As chronic inflammation plays a vital role in tumor initiation, progression, and metastases, we hypothesized that *p53*inactivation in the TME favors tumorigenesis by promoting inflammation. To test our hypothesis and elucidate the cellular and molecular mechanisms by which *p53* inactivation augments pro-inflammation and tumor progression, we compared the growth of subcutaneously inoculated B16F1 melanoma with a functional p53 in *p53null* and WT mice. As expected, tumor growth in *p53null*mice was greatly accelerated. Remarkably, the accelerated tumor growth in *p53null* hosts was associated with an extensive expansion of stromal populations, including various myeloid populations, as well as non-hematopoietic reticular fibroblastic cells (FRC) reminiscent of stromal cells of the secondary lymphoid organs (SLO), both within the TME and the secondary lymphoid tissues. Further cellular and molecular analyses revealed that these CD106hiCD54+GP38+Sca-1lo/- FRCs, especially from *p53null* hosts, expressed high levels of pro-inflammatory cytokines/chemokines and immunosuppressive mediators that supported the survival and proliferation of various myeloid populations, including CD11b+Gr-1+ myeloid-derived suppressor cells (MDSCs). Together, our results suggest that *p53null* stroma is highly immunosuppressive, which modulates host immune-microenvironment via cytokine/chemokine and stroma-immune cell interaction and promotes tumor progression. This study underscores the immunological function of p53 in tumor suppression and broadens our appreciation of the p53 as a guardian and gatekeeper not only via inducing apoptosis and cellular senescence, but also via regulating the immunological microenvironment.

